# A Selective Cucurbit[8]uril‐Peptide Beacon Ensemble for the Ratiometric Fluorescence Detection of Peptides

**DOI:** 10.1002/chem.201901037

**Published:** 2019-09-17

**Authors:** Debabrata Maity, Khaleel I. Assaf, Wilhelm Sicking, Christoph Hirschhäuser, Werner M. Nau, Carsten Schmuck

**Affiliations:** ^1^ Institute of Organic Chemistry University of Duisburg–Essen Universitatsstrasse 7 45117 Essen Germany; ^2^ Department of Life Sciences and Chemistry Jacobs University Bremen Campus Ring 1 28759 Bremen Germany

**Keywords:** cucurbit[*n*]uril, fluorescence, insulin, peptide beacon, supramolecular chemistry

## Abstract

A convenient supramolecular strategy for constructing a ratiometric fluorescent chemosensing ensemble, consisting of a macrocyclic host (cucurbit[8]uril CB[8]), and a pyrene‐tagged amphiphilic peptide beacon (AP 1), is reported. AP 1 unfolds upon encapsulation of the pyrene termini into the hydrophobic CB[8] cavity. This changes pyrene excimer to monomer emission. Substrates with higher affinity for the CB[8] cavity can displace AP 1 from the ensemble. The released AP 1 folds again to form a pyrene excimer, which allows for the ratiometric fluorescence monitoring of the substrate. In this report, the ensemble capacity for ratiometric fluorescence monitoring of biological substrates, such as amino acid derivatives, specific peptides, and proteins, in aqueous media is demonstrated.

## Graphical abstract

**A switching chemosensing ensemble** was obtained by combining a macrocyclic host, cucurbit[8]uril (CB[8]), and a pyrene‐tagged amphiphilic peptide beacon (AP 1) for excellent ratiometric fluorescent recognition of biological substrates in aqueous media. The pyrene termini of the flexible AP 1 compete with substrates for encapsulation into the CB[8] cavity which changes pyrene excimer/monomer emission.

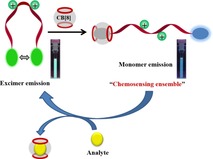

Supramolecular chemistry based indicator displacement assays (IDAs) that are based on synthetic receptors, particularly macrocyclic hosts, for molecular sensing have gained considerable interest over the last few decades. The sensing principle of IDAs is based on the competitive binding between an indicator and an analyte for a host (receptor).^1^ Initially, the indicator is bound to a receptor to form a chemosensing ensemble. Subsequently, a target analyte with high affinity towards the host displaces the indicator from the sensing ensemble. The release of the indicator results in a change of its photophysical properties, for example, of its fluorescence.^2^ Thus, in IDAs, the challenging chemical synthesis of chemosensors containing the recognition unit and a covalently linked signaling unit can be avoided. Furthermore, the binding property of the receptor is also conserved. Macrocycles such as cucurbit[*n*]urils (CB[*n*]) are capable of binding and recognizing guest molecules, such as fluorescent dyes in aqueous media.^3^ The fluorescence of the dye is generally enhanced upon encapsulation into a more hydrophobic, solvent‐protected environment of the CB[*n*] cavity. The target analyte undergoes a displacement reaction with the CB[*n*]⋅dye complex, which releases the dye into the polar medium. This results in a detectable fluorescence “switch‐off” signal. Several fluorescent guests for CB[*n*], and myriad cases of IDA‐based detection of optically inactive analytes have been reported, mostly with quenching of a fluorescence signal.^4^ There are many disadvantages of using simple fluorescence switching and, in particular, fluorescence signal switch‐off for signaling. For example, impurities in the sample may quench fluorescence or photobleaching of the fluorophore may occur.^5^ To remedy this shortcoming, ratiometric sensing and imaging techniques have become popular, as they avoid these issues by observing dual shifts in emission wavelengths of the fluorophores or by comparing two emission intensities of a fluorophore pair instead of measuring a single quantity.^6^ However, CB[*n*]‐based ratiometric fluorescent detection of analytes is rare.^7^ A diaminoalkyl‐functionalized carbazole dye with CB[6] was reported earlier for ratiometric fluorescence monitoring of l‐lysine to cadaverine conversion by using lysine decarboxylase.^8^ A CB[7]‐cyanine‐3 conjugate was used in combination with its fluorescence resonance energy transfer (FRET) pair (adamantane ammonium conjugated cyanine‐5) to detect vesicle fusion.^9^

Recently, we have successfully employed a pyrene‐functionalized cationic peptide‐based molecular beacon for the ratiometric fluorescent detection of nucleic acids and heparin.^10^ The peptide beacons were designed in such a way that they underwent a conformational change from the folded to the open form (or vice versa) upon binding with biomolecules. These conformational changes are accompanied by switching of fluorescence from either excimer to monomer or vice versa. Here, we present a pyrene‐based amphiphilic peptide beacon (AP 1) and cucurbit[8]uril (CB[8]) conjugate (AP 1⋅CB[8]) for advantageous ratiometric fluorescence applications (Scheme 1). AP 1 has two symmetric amphiphilic peptidic arms attached via the C‐terminus to a central lysine spacer. Each peptidic arm contains a cationic lysine as a positively charged head group. A pyrene moiety is attached through a lipophilic γ‐aminobutyric acid linker to the end of each arm providing amphiphilicity. AP 1 is synthesized by conventional microwave‐assisted solid‐phase peptide synthesis. The Schmuck group mostly focused on developing receptors and chemosensors for substrates of biological importance.^11^ Amino acids, short peptides, and proteins were selected as potential binders for CB[8] to be investigated by using a ratiometric fluorescence‐based technique.

**Scheme 1:**
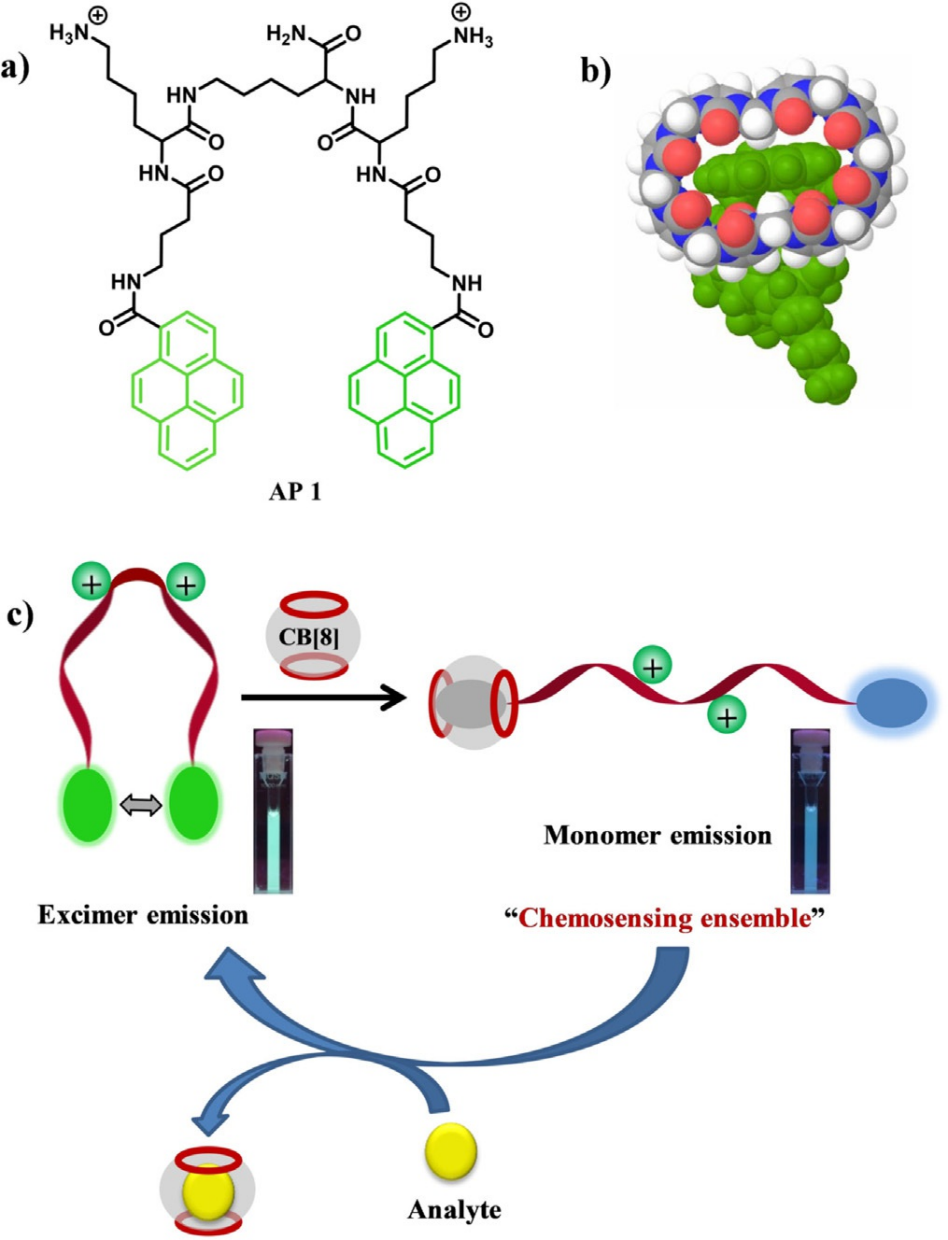
(a) Molecular structure of AP 1. (b) Possible binding mode of pyrene termini of AP 1 to CB[8] according to molecular modeling. (c) Cartoon representation of ratiometric fluorescence detection application by using AP 1 and CB[8] conjugate (the photographs show the corresponding cuvettes under UV light).

The emission spectrum of free AP 1 (5 μm) features a strong band at 505 nm and a significantly weaker one at 385 nm upon 340 nm excitation in aqueous media (10 mm HEPES, pH 7.2) (Figure 1). The band at 505 nm arises from a pyrene excimer, whereas the band at 385 nm is the characteristic emission of a pyrene monomer. This indicates that amphiphilic AP 1 is mostly present in its folded form in solution.

**Figure 1:**
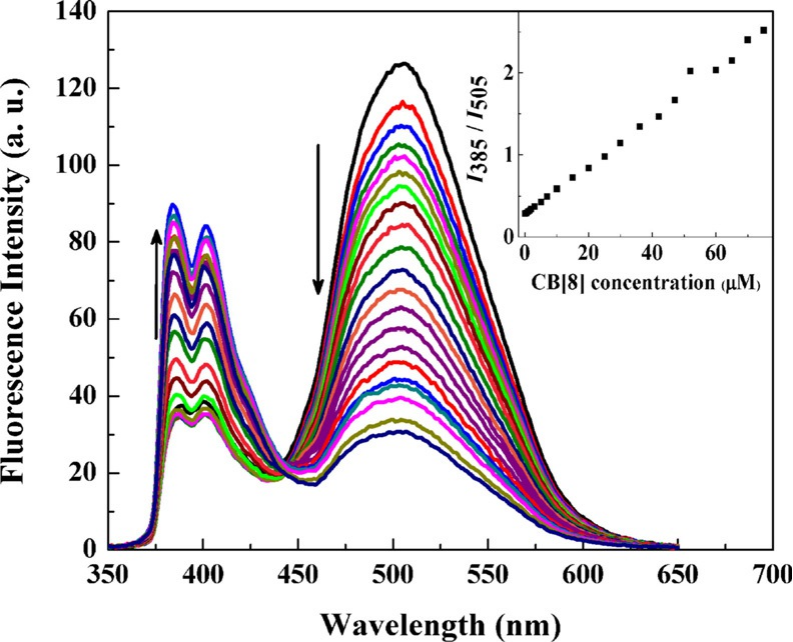
Ratiometric fluorescence emission spectra of AP 1 (5 μm) upon addition of CB[8] (0–75 μm) (*λ*_ex_=340 nm) in 10 mm HEPES buffer, pH 7.4. Inset: Ratiometric fluorescence change during titration of AP 1 by CB[8].

However, the excimer emission decreases, and the monomer emission increases, concomitantly upon CB[8] (0–50 μm) addition (Figure 1). A clear isosbestic point is observed at 443 nm, which indicates formation of new species. After addition of more than 50 μm CB[8], both monomer and excimer emission decrease, which is presumably due to the formation of a 1:2 complex. Ratiometric fluorescence intensity (*I_385_/I_505_*) is still increased further after addition of 60 μm CB[8] as the fluorescence intensity at 505 nm is reduced at a higher rate than at 385 nm (Figure S1, Supporting Information). To investigate the interesting fluorescence property of AP 1 in the presence of CB[8], we synthesized an amphiphilic control peptide (CP) with a structure similar to a single peptide arm of AP 1, in which a pyrene moiety is connected to a cationic lysine through a lipophilic γ‐aminobutyric acid linker. Emission spectra of free CP (5 μm) feature a strong monomer band at 385 nm, the intensity of which is decreased upon addition of CB[8] (Figure S2, Supporting Information). There is no pyrene excimer formation during this CB[8] titration. In a recent report, CB[8] decreased intrinsic pyrene monomer emission of a pyrene‐functionalized peptide.^12^ Quenching of monomer emission is probably due to an electron transfer from CB[8] to pyrene.^13^ We have performed molecular modeling that shows that each CB[8] can accommodate a maximum of one pyrene moiety in its cavity (Scheme 1).

Therefore, CB[8] complexation probably quenches pyrene monomer fluorescence. During sequential addition of CB[8] to AP 1, one pyrene of the peptide beacon is slowly complexed with CB[8], whereas the other pyrene of the pair remains free. This phenomenon is accountable for enhancing pyrene monomer emission and decreasing excimer emission. Maximum ratiometric fluorescent behavior of 5 μm AP 1 is observed up to addition of 50 μm CB[8]. At this concentration, one pyrene moiety of most peptide beacons is complexed by CB[8], whereas the other (“dangling”) pyrene moieties are free. However, after addition of more than 50 μm CB[8] to 5 μm AP 1, the remaining free pyrene moieties of AP 1 are gradually complexed with CB[8] resulting in decreased monomer and excimer emission. Accordingly, for further fluorescence studies, we have chosen 5 μm AP 1 and 50 μm CB[8] as an ideal concentration ratio for our chemosensing ensemble to generate maximum ratiometric fluorescence output. Mass spectrometric analysis of the AP 1 and CB[8] mixture also confirmed a 1:2 complex (Figure S3, Supporting Information). Inclusion of pyrene into CB[8] has been previously noted;^14^ for AP 1 as the guest, the complexation was confirmed by ^1^H NMR spectroscopy in D_2_O (Figure S4, Supporting Information). The addition of CB[8] to a solution of AP 1 resulted in a downfield shift of the aromatic pyrene peaks. A similar downfield shift of aromatic peaks was observed during the formation of a perylene bis(diimide)‐CB[8] complex.3d Generally, encapsulation of hydrophobic moieties inside the cavity of CB[8] leads to an upfield shift, but also disarrays pyrene stacking, which leads to a competing downfield shift. Notably, the AP 1 ^1^H NMR signals were very broad, which might indicate that CB[8] shuttles between the two pyrene moieties.

Previous reports have indicated that CB[8] prefers to bind to N‐terminal aromatic amino acid (e.g., Trp and Phe) residues present in peptides and proteins.^15^ This specific recognition is simultaneously governed by formation of an inclusion complex of hydrophobic aromatic side chains within the nonpolar cavity of the cucurbituril and electrostatic attraction of the cationic N‐terminal ammonium group with the negative dipoles of the carbonyl groups present at the portals of CB[8].

Therefore, we screened a series of tryptophan derivatives as preliminary guests for a ratiometric fluorescence study by using the AP 1⋅CB[8] chemosensing ensemble (Figure S5, Supporting Information). Zwitterionic tryptophan (Trp), cationic tryptophan methyl ester (TrpOMe), tryptamine (TrpA), anionic *N*‐acetyl tryptophan (*N*‐AcTrp), and indole propionic acid (IPA) were studied. As expected, the monomer emission (*I*_385_) of the 5 μm AP 1 and 50 μm CB[8] mixture decreases, and the excimer emission (*I*_505_) increases concomitantly upon addition of TrpOMe (Figure 2). The emission intensity ratio (*I*_505_/*I*_*3*85_) gradually increases from 0.42 to 1.89 and becomes almost constant after addition of 50 μm TrpOMe. This indicates 1:1 complexation of TrpOMe with CB[8]. Such a ratiometric fluorescence response was not observed in the absence of CB[8] (Figure S6, Supporting Information). Following displacement by TrpOMe, free AP 1 again folds back to form the pyrene excimer in the aqueous medium. A similar ratiometric fluorescence change was also observed for tryptamine addition (Figure 2 and Figure S7 in the Supporting Information). Only a very weak ratiometric fluorescence change was observed for IPA but not for *N*‐AcTrp (Figure 2 and Figure S7 in the Supporting Information). IPA and *N*‐AcTrp lack the attractive positive charges required for molecular recognition by CB[8] but rather contain repulsive negative charges. Trp, having both positive and negative charges, showed fluorescence quenching of both monomer and excimer emission, but failed to afford a ratiometric response (Figure 2 and Figure S7 in the Supporting Information).

**Figure 2:**
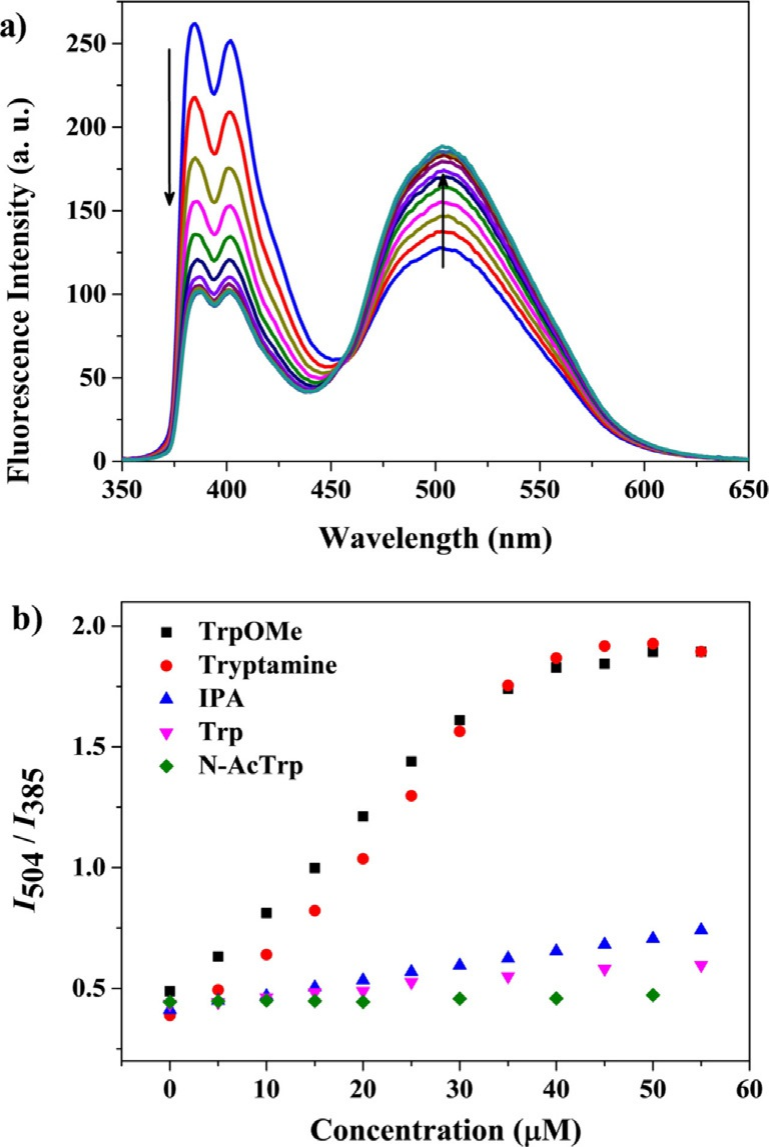
a) Ratiometric fluorescence emission spectra of a 5 μm AP 1 and 50 μm CB[8] mixture upon addition of TrpOMe (0–55 μm) (*λ*_ex_=340 nm) in 10 mm HEPES buffer, pH 7.4. b) Ratiometric fluorescent responses of a 5 μm AP 1 and 50 μm CB[8] mixture upon addition of different tryptophan derivatives (0–55 μm) (*λ*_ex_=340 nm) in 10 mm HEPES buffer, pH 7.4.

The remarkably strong ratiometric fluorescence response of TrpOMe encouraged us to study some other significant amino acid methyl ester derivatives (Figures S8 and S9, Supporting Information). Methyl esters of hydrophobic amino acids like phenylalanine (PheOMe) and leucine (LeuOMe) increase the *I*_505_/*I*_385_ values from 0.42 to 1.80 and 1.68, respectively. *I*_505_/*I*_385_ also increases from 0.42 to 1.58 and 1.38 for the amphiphilic lysine (LysOMe) and arginine (ArgOMe) methyl esters, respectively. The electron‐rich tyrosine methyl ester (TyrOMe) increases *I*_505_/*I*_385_ to a lesser extent (from 0.42 to 1.25). As expected, the negatively charged methyl esters of aspartic acid (AspOMe) and glutamic acid (GluOMe) failed to show any emission intensity ratio. Therefore, displacement of the pyrene termini from the CB[8] cavity by amino acid methyl ester derivative guests depends on their hydrophobic nature.

The ratiometric fluorescence study with different tryptophan derivatives clearly demonstrates that CB[8] binds N‐terminal aromatic residues with higher affinity than C‐terminal or internal aromatic residues, thereby providing a basis for the ratiometric fluorescence detection of specific peptides. To confirm this hypothesis, we synthesized two short peptides: FGG and GFG containing hydrophobic phenylalanine residues for comparing the ratiometric fluorescence response by using the 5 μm AP 1 and 50 μm CB[8] chemosensing ensemble. FGG titrations showed an interesting fluorescent behavior (Figure 3); addition of 50 μm FGG showed weak quenching of pyrene excimer emission and little interference with pyrene monomer fluorescence. The expected ratiometric fluorescence behavior is observed when more than 50 μm FGG is added. Actually, CB[8] can accommodate two N‐terminal hydrophobic phenylalanine residues of two FGG peptides in its cavity, as shown nicely by Urbach and co‐workers by means of crystal structures and ITC data.^16^

**Figure 3:**
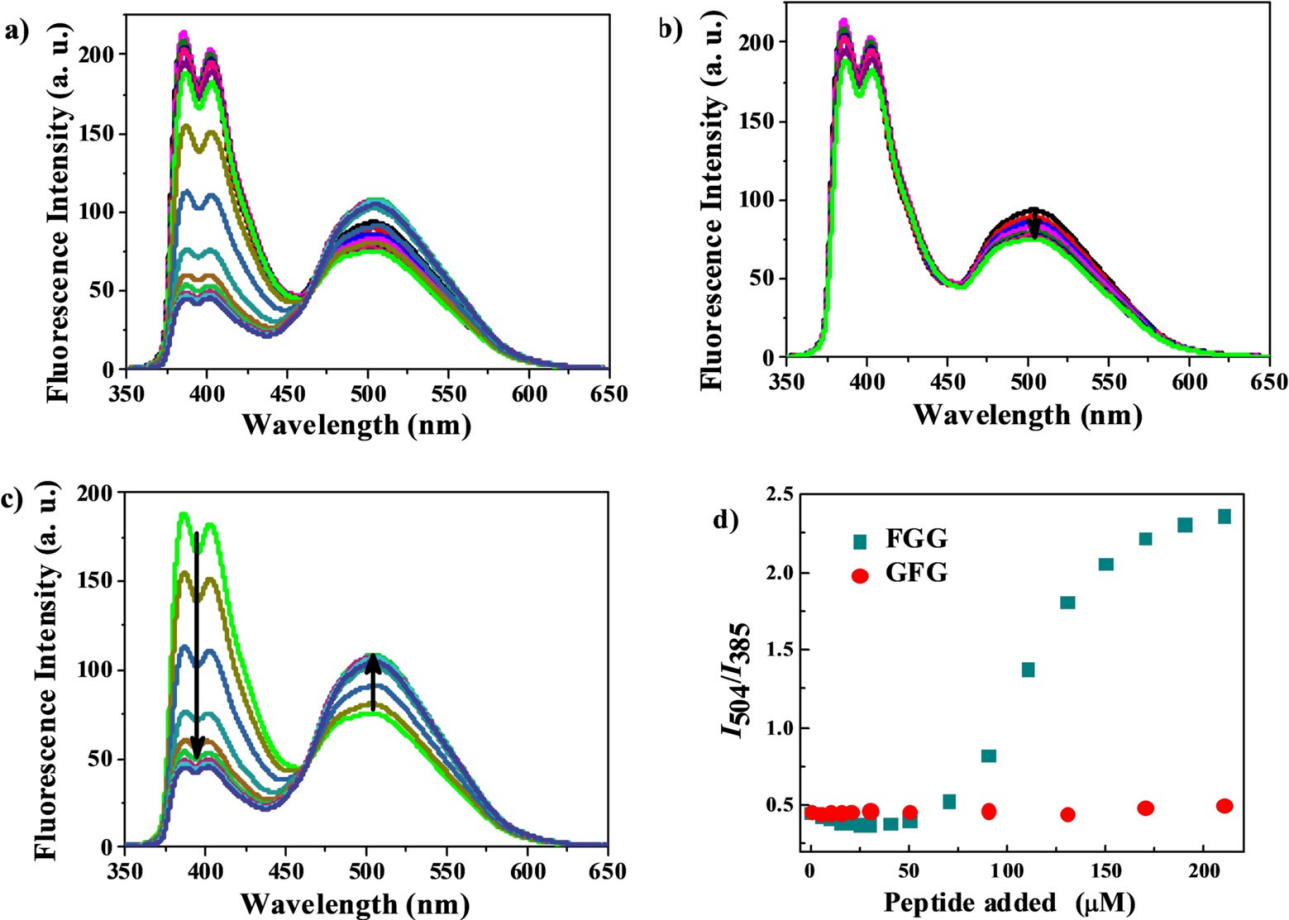
a) Full fluorescence titration of a 5 μm AP 1 and 50 μm CB[8] mixture upon addition of FGG peptide (0–210 μm) (*λ*_ex_=340 nm) in 10 mm HEPES buffer, pH 7.4. b) Initial stage of titration upon FGG addition (0–50 μm) and c) final stage of titration upon FGG addition (50–210 μm). d) Ratiometric fluorescence comparison between the peptides FGG and GFG by using the 5 μm AP 1 and 50 μm CB[8] chemosensing ensemble.

In this work, we also observed that binding of two FGG peptides occurs in a stepwise manner. Initially, the N‐terminal hydrophobic phenylalanine residue of one equivalent FGG is encapsulated into the CB[8], in which it stays along with the pyrene termini of AP 1. However, addition of more than one equivalent of FGG removes the pyrene terminus of AP 1 from the CB[8] cavity. Released AP 1 again folds to form a pyrene excimer. Molecular modeling also showed that the CB[8] cavity can accommodate one pyrene terminus of AP 1 and one N‐terminal phenylalanine residue of FGG together (Figure S10, Supporting Information). Addition of FGG to AP 1 in the absence of CB[8] showed quenching of both monomer and excimer fluorescence but no ratiometric response (Figure S11, Supporting Information). GFG titration with a 5 μm AP 1 and 50 μm CB[8] mixture failed to show any ratiometric behavior. Instead, it weakly quenched both monomer and excimer fluorescence (Figure S12, Supporting Information). Therefore, the AP 1⋅CB[8] ensemble selectively recognizes peptides containing N‐terminal aromatic amino acids, such as phenylalanine, with noticeable ratiometric fluorescence response.

We further used our system for ratiometric fluorescence detection of insulin, inspired by the work of Urbach and co‐workers.4b They showed that cucurbit[7]uril binds to the N‐terminal phenylalanine (Phe) of human insulin more strongly than to other proteins lacking an N‐terminal aromatic residue. Insulin has a rare Phe residue at the N‐terminus of its B‐chain (i.e., Phe^B1^). Titration of insulin by using 5 μm AP 1 and 50 μm CB[8] showed ratiometric fluorescence behavior but in a reverse manner. The full ratiometric fluorescent studies are shown in Figure 4 a. Initially, addition of insulin increases further pyrene monomer emission while quenching the weak pyrene excimer emission (Figure 4 b). However, addition of insulin at a concentration higher than 50 μm started to quench pyrene monomer emission but slightly increased pyrene excimer emission (Figure 4 c). The complete ratiometric profile is shown in Figure 4 d. To understand this phenomenon, we performed molecular modelling. It showed that one CB[8] cavity can accommodate one pyrene terminus of AP 1 and part of the Phe residue at the N‐terminus of the insulin B‐chain together (Figure S13, Supporting Information). Therefore, we claim that the N‐terminal Phe residue of insulin and one pyrene terminus of AP 1 are encapsulated together inside the CB[8] cavity at the initial stage of insulin titration (0–50 μm). The large proteohormonal structure of complexed insulin probably changes the microenvironment around the other pyrene terminus of the complexed AP 1. This results in an enhancement of pyrene monomer emission. Further addition of insulin (>50 μm) displaces the pyrene‐tagged AP 1 from CB[8] in the solution. This results in quenching of pyrene monomer emission. The released AP 1 fails to form a proper pyrene excimer, which results in minimal pyrene excimer emission, as at this stage the solution is over‐crowded with the large insulin structure.

**Figure 4:**
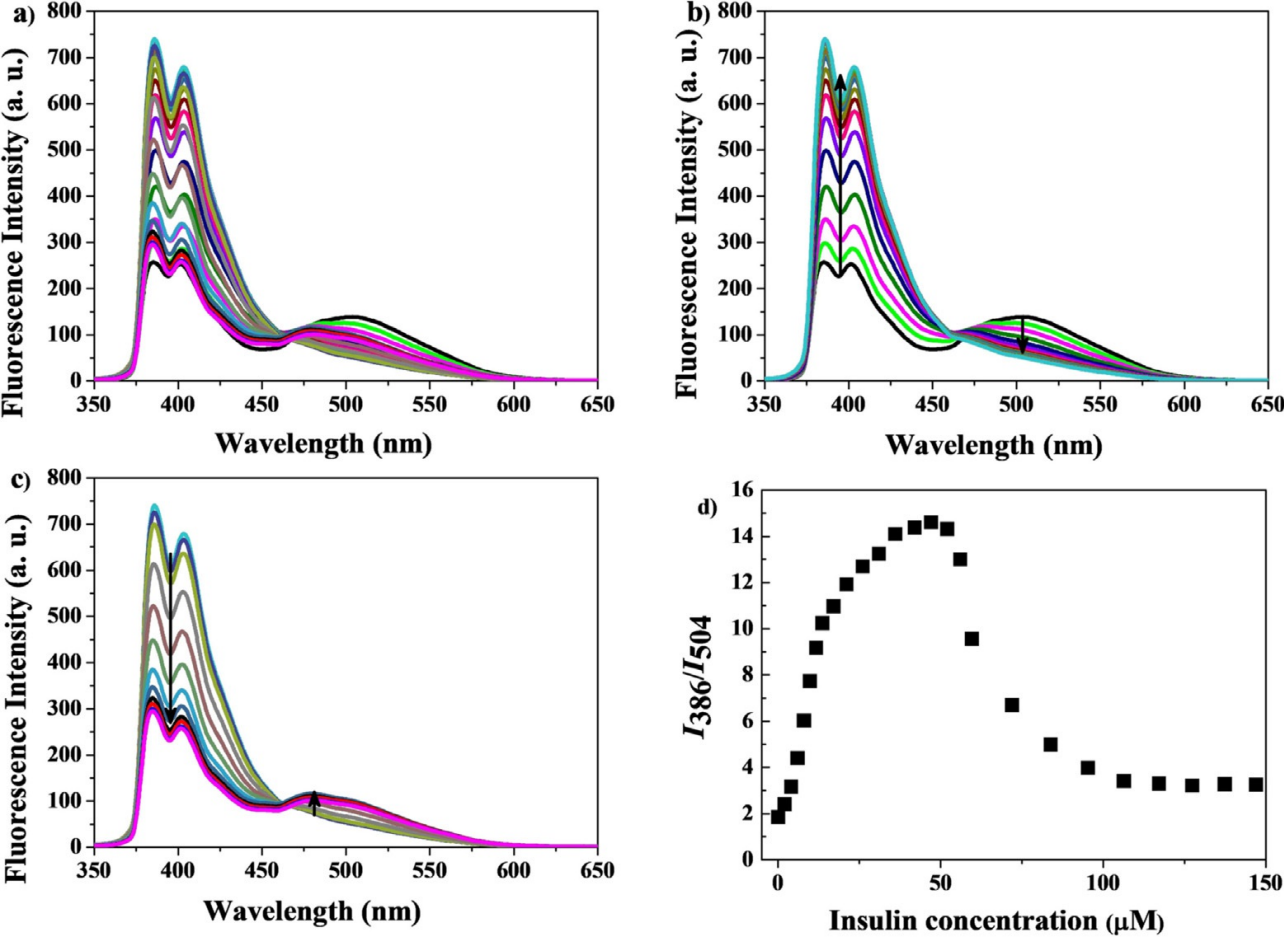
a) Full fluorescence titration of a 5 μm AP 1 and 50 μm CB[8] mixture upon addition of insulin (0–147 μm) (*λ*_ex_=340 nm) in 10 mm HEPES buffer, pH 7.4. b) Initial stage of titration upon insulin addition (0–50 μm) and c) final stage of titration upon insulin addition (50–147 μm). d) Ratiometric fluorescence response (*I*_386_/*I*_504_) of insulin (0–147 μm) addition to the 5 μm AP 1 and 50 μm CB[8] chemosensing ensemble.

Finally, we checked the ratiometric fluorescence responses of the AP 1⋅CB[8] ensemble towards other commercially available blood proteins, including bovine serum albumin (BSA), human immunoglobulin G (IgG), and bovine carbonic anhydrase (BCA). These proteins failed to show any significant ratiometric behavior as they lack N‐terminal aromatic amino acid residues (Figure 5 a). Only BSA showed a weak response because it quenches pyrene excimer emission to some extent (Figure S14, Supporting Information). AP 1⋅CB[8] selectively detects insulin even in the presence of the other proteins (Figure 5 b and Figure S17 in the Supporting Information). Therefore, selective ratiometric fluorescent detection of insulin in aqueous media by the AP 1⋅CB[8] conjugate was successfully demonstrated.

**Figure 5:**
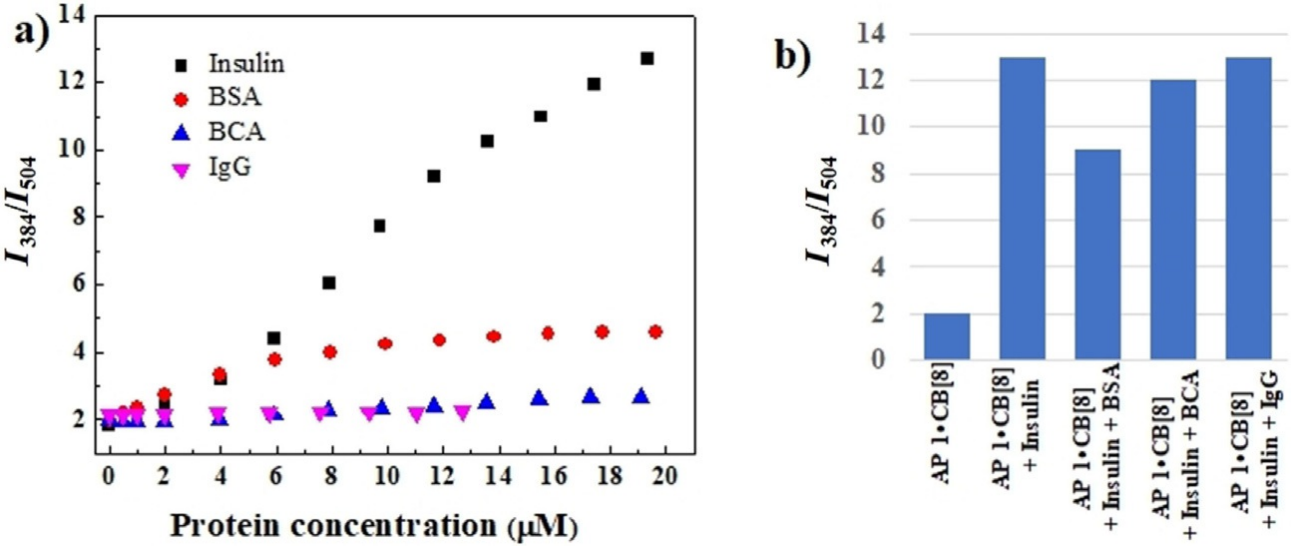
a) Ratiometric fluorescence responses of 5 μm AP 1 and 50 μm CB[8] towards different blood proteins (λ_ex_=340 nm) in 10 mm HEPES buffer, pH 7.4. b) Competitive ratiometric fluorescence responses for detection of insulin (20 μm) by using 5 μm AP 1 and 50 μm CB[8] in the presence of BSA, BCA, and IgG, respectively (20 μm).

In conclusion, we have presented a supramolecular peptide beacon and cucurbit[8]uril chemosensing ensemble (AP 1⋅CB[8]) for ratiometric fluorescence detection applications. The pyrene‐tagged amphiphilic peptide beacon (AP 1) unfolds upon encapsulation of a single pyrene terminus into the hydrophobic CB[8] cavity resulting in changes in emission from excimer to monomer. However, the AP 1⋅CB[8] ensemble displays concentration‐dependent monomer to excimer ratiometric fluorescence behavior because guests displace AP 1 from the CB[8] cavity. The conjugation of the peptide beacon does not alter the binding property of CB[8]. By using ratiometric fluorescence detection, the AP 1⋅CB[8] conjugate successfully differentiates methyl ester derivatives of different amino acids based on their CB[8] binding affinity. The AP 1⋅CB[8] ensemble is also highly selective for aromatic amino acid residues present at the N‐terminus of a peptide. Finally, this system was used for the selective ratiometric fluorescent detection of insulin. This kind of pyrene‐tagged amphiphilic peptide beacon conjugated to CB[8] could serve as a conceptual blueprint for ratiometric fluorescence monitoring of several CB[8] macrocycle‐associated applications in drug delivery, catalysis, and other applications.

## Conflict of interest

The authors declare no conflict of interest.

## Supporting information

As a service to our authors and readers, this journal provides supporting information supplied by the authors. Such materials are peer reviewed and may be re‐organized for online delivery, but are not copy‐edited or typeset. Technical support issues arising from supporting information (other than missing files) should be addressed to the authors.

Supplementary
