# Supplementary material for: A Selective Cucurbit[8]uril‐Peptide Beacon Ensemble for the Ratiometric Fluorescence Detection of Peptides
Source: Chemistry. 2019 Sep 17;25(57):13088–93. doi: 10.1002/chem.201901037 (PMC6856807; doi:10.1002/chem.201901037)
Supplement: Supplementary file 1 — Supplementary [file CHEM-25-13088-s001.pdf]

CHEMISTRY
A European Journal

Supporting Information

# A Selective Cucurbit[8]uril-Peptide Beacon Ensemble for the Ratiometric Fluorescence Detection of Peptides

Debabrata Maity,[a] Khaleel I. Assaf,[b] Wilhelm Sicking,[a] Christoph Hirschhäuser,*[a] Werner M. Nau,[b] and Carsten Schmuck+[a]

chem_201901037_sm_miscellaneous_information.pdf

# Supporting Information

# A Selective Cucurbit[8]uril-Peptide Beacon Ensemble for Ratiometric Fluorescence Detection of Peptides

Debabrata Maity, Khaleel I. Assaf, Wilhelm Sicking, Christoph Hirschhäuser*, Werner M. Nau, and Carsten Schmuck[†]

**Contents:**

1. Materials and methods S2
2. General procedure for the synthesis of peptide S2-S3
3. Synthesis of peptide **AP 1, CP** and their characterization S3-S9
4. Additional experiments S10-S20

[a] Dr. D. Maity, W. Sicking, Dr. Christoph Hirschhäuser, Prof. C. Schmuck[†]
Institute of Organic Chemistry, University of Duisburg-Essen, Universitatsstrasse 7, 45117 Essen, Germany
E-mail: Christoph.Hirschhäuser@uni-due.de
[b] Dr. K. I. Assaf, Prof. W. M. Nau
Department of Life Sciences and Chemistry, Jacobs University Bremen Campus Ring 1, 28759 Bremen (Germany)

Supporting information for this article is given via a link at the end of the document.

## 1. Materials and methods:

Solvents were dried and distilled before use. Millipore water was obtained with a Micropure apparatus from TKA. Lyophilization was carried out with an Alpha 1-4 2D plus freeze drying apparatus from Christ. Analytical TLC was carried out on $SiO_2$ aluminium foils ALUGRAM SIL G/$UV_{254}$ from Macherey-Nagel. Reversed phase column chromatography was done with an Armen Instrument Spot Flash Liquid Chromatography MPLC apparatus with RediSep C-18 Reversed-Phase columns. The purity of the compounds was determined with the help of an HPLC apparatus from Dionex containing the following components: P680 HPLC pump, ASI-100 Automated Sample Injector and UVD 340U detector. A Supelcosil TM LC-18 column (25 cm × 4.6 mm, 5 µm) from Supelco or a YMC ODS-A column (15 cm × 3.0 mm, 5 µm) was utilized. Ultrapure water and HPLC-grade solvents were used as eluents. Detection was achieved with the help of a UV detector. $^{1}$H- and $^{13}$C-NMR spectra were recorded on DRX 500 MHz spectrometer from Bruker at ambient temperature. The chemical shifts are reported in parts per million (ppm) relative to the deuterated solvent DMSO-$d_6$ and $D_2O$. All mass spectra were received by using a Bruker BioTOF III. Determination of pH values was carried out with a pH-Meter 766 Calimatic from Knick. Fluorescence spectra were obtained with a Varian Cary Eclipse spectrometer. For calculation, software used: MacroModel, V11.4, Schrodinger, LLC and method used: OPLS2005/Water.

## 2. General procedure for the synthesis of the peptide:

**Fmoc Removal:** The Fmoc protecting group was cleavage by treatment with 20% piperidine in DMF (2×6 mL, 5 min each) under microwave radio condition (20 W, 50±5 °C, 5 min). Then, the resin was washed 3×8 mL with DMF, 3×8 mL with DCM, 3×8 mL with DMF (ca. 5 min each) to remove the last traces of piperidine. A positive Kaiser test confirmed the cleavage of the Fmoc group and the presence of a free amino function.

**Standard Fmoc solid phase peptide synthesis techniques (SPPS):** Each amino acid was attached using 0.6 mmol/g loading Fmoc Rink amide resin under microwave radio (20 W, 60±5 °C, 20 min) Then, the resin was washed 3×8 mL with DMF, 3×8 mL with DCM, 3×8 mL with DMF (ca. 5 min each) to remove the last traces of the amino acid. A negative Kaiser test confirmed the attachment of the corresponding amino acid.

**Cleavage from the Resin:** Cleavage of the product from the resin was achieved by treatment with a mixture of TFA/$H_2O$/triisopropylsilane (95:2.5:2.5) for 3 h. The yellow cleavage mixture was collected by filtration and the resin was washed twice with pure TFA (6 mL). The filtrates were combined and concentrated under vacuum to obtain an oily residue. The peptide was precipitated by adding dry diethyl ether to the oil, following by centrifugation of the mixture. The precipitate was dissolved in water (25 mL), and the mixture was freeze dried in vacuum. The resulting solid was purified by MPLC on C18 reversed phase silica gel (MeOH/water, 0.1 % TFA). Then the product was dissolved three times in $HCl_{aq}$ (0.1 N) plus one time in water and consequently freeze dried to obtain white solid.

Purity of the peptides was checked by HPLC on a RP18-column using water/MeOH (with 0.05% TFA) as solvent.

### 3. Synthesis of AP 1 and CP and their characterization:

**Synthesis of AP 1:** Rink amide 4-methylbenzhydrylamine (MBHA) resin (0.6 mmol/g) was swollen in DCM and Fmoc-Lys(Fmoc)-OH was attached as a spacer to the solid support under argon atmosphere using PyBOP as a coupling reagent in 5% DIPEA/DMF (three equivalents of each reactant). With the help of a single-mode microwave, the reaction mixture was irradiated for 20 min at 20 W and allowed to reach a maximum temperature of 60 °C. The coupling step was repeated to assure complete conversion of all accessible amino groups on the resin. After deprotection of the Fmoc group with 20% piperidine/DMF (1+5 min, 20 W, max. 60 °C), Fmoc-Lys(Boc)-OH, Fmoc-GABA-OH and pyrene carboxylic acid were coupled similarly using six equivalents of each reactant. Finally, the resin was thoroughly washed and dried and the receptor was cleaved from the solid support; the Boc-protected side chains were deprotected at the same time without microwave irradiation by utilizing a cleavage mixture composed of trifluoroacetic acid (TFA)/water/triisopropylsilane (TIS) (95:2.5:2.5). After purification by means of precipitation and reversed-phase medium-pressure liquid chromatography (MPLC) with 0.1% TFA in $H_2O$/MeOH and consequent anion exchange, probe AP 1 was obtained as hydrochloride salt.

**Peptide AP 1:**

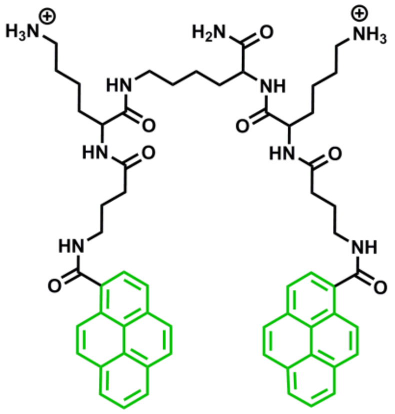

**Peptide AP 1** (55.6 mg, 54 µmol, Yield 45%, purity HPLC 97%). $^{1}$H NMR (500 MHz, DMSO-$d_6$): δ[PPM] 1.22-1.37 (m, 8H), 1.52- 1.67 (m, 10H), 1.84-1.90 (m, 4H), 2.31-2.36 (dd, $J$ = 7.7 Hz, 8.5 Hz, 4H), 2.73-2.76 (m, 4H), 2.95-3.06 (m, 2H). 3.34-3.49 (m, 4H), 4.12-4.29 (m, 3H), 7.03 (s, 1H), 7.34 (s, 1H), 7.90-7.95 (m, 7H), 8.00 (t, $J$ = 5.5 Hz, 1H), 8.08-8.09 (d, $J$ = 8.3 Hz, 1H), 8.10-8.13 (t, $J$ = 7.6 Hz, 2H), 8.15-8.13 (dd, $J$ = 6.1 Hz, 1.7 Hz, 2H), 8.18-8.20 (m, 1H), 8.21-8.26 (m, 6H), 8.31-8.36 (m, 6H), 8.48-8.50 (dd, $J$ = 7.6 Hz, 1.6 Hz, 2H), 8.74-8.77 (q, $J$ = 5.5 Hz, 2H). $^{13}$C NMR (125.8 MHz, DMSO-$d_6$): δ[PPM] 22.26, 22.38, 22.74, 25.48, 26.52, 26.56, 28.70, 31.11, 31.48, 31.54, 32.76, 38.37, 38.51, 39.76, 39.93, 52.39, 52.61, 123.63, 123.77, 124.36, 124.67, 125.24, 125.56, 125.76, 126.54, 127.18, 127.72, 128.05, 128.21, 130.17, 130.69, 131.50, 132.04, 168.90, 171.48, 171.59, 171.89, 172.25, 173.65. MALDI-TOF MS: cald 1050.5212 for $C_{60}H_{69}N_9O_7$ + $Na^+$, found 1050.3062.

**RP-HPLC chromatogram of Peptide AP 1:**

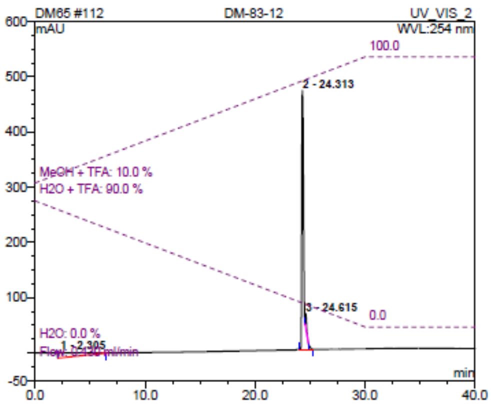



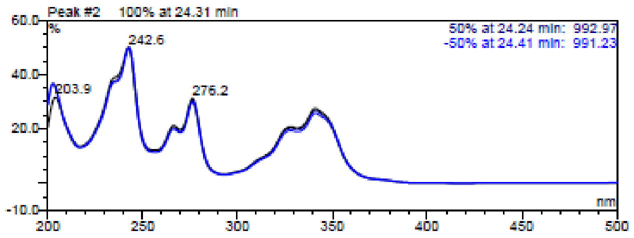

# 500 MHz $^1$H NMR Bruker AVANCE 500 (DRX)

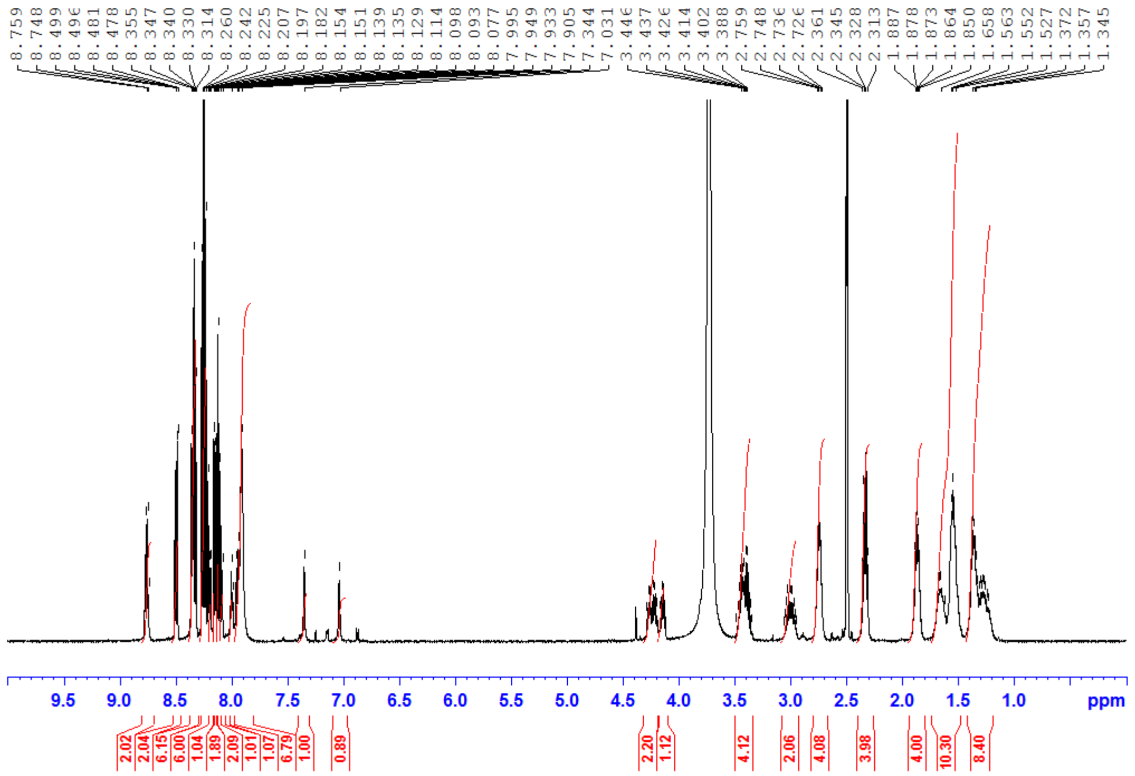


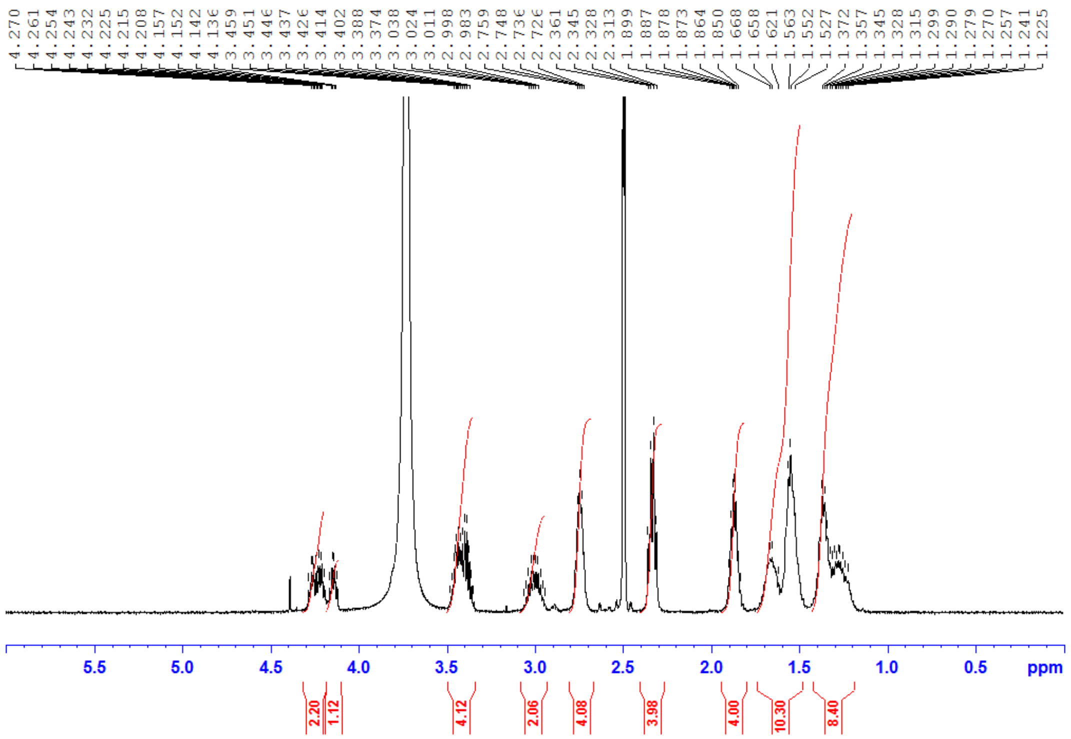

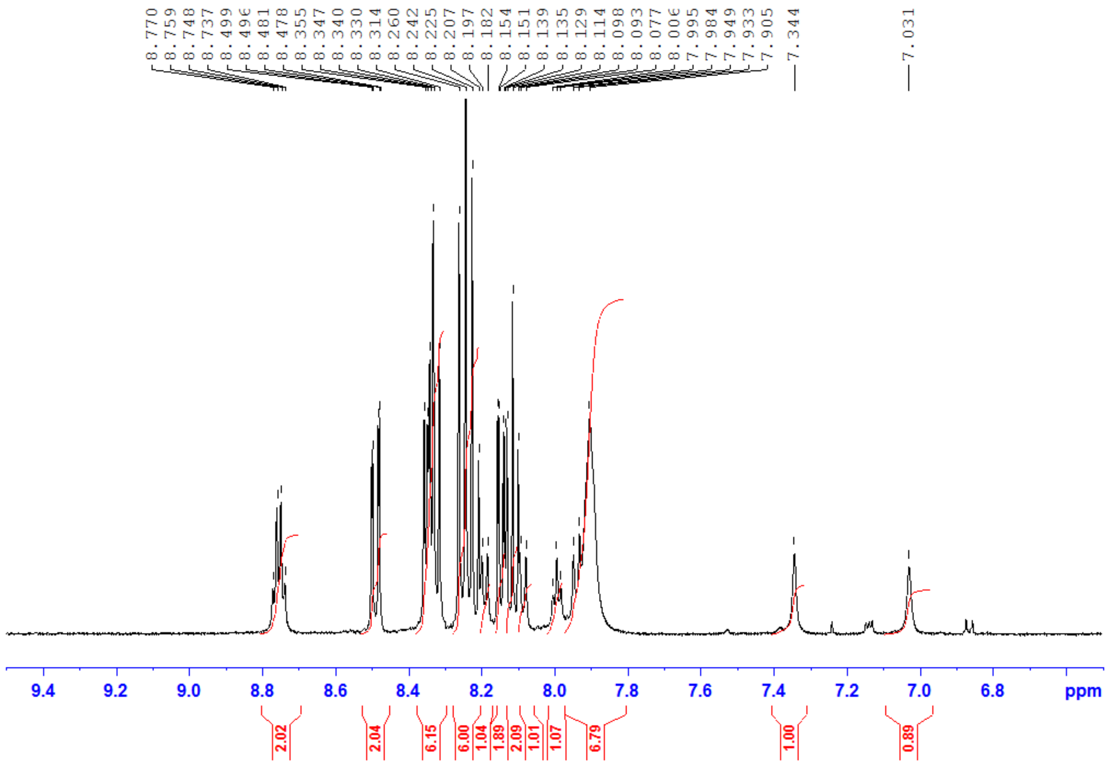


**125.8 MHz $^{13}$C NMR Bruker AVANCE 500 (DRX)**

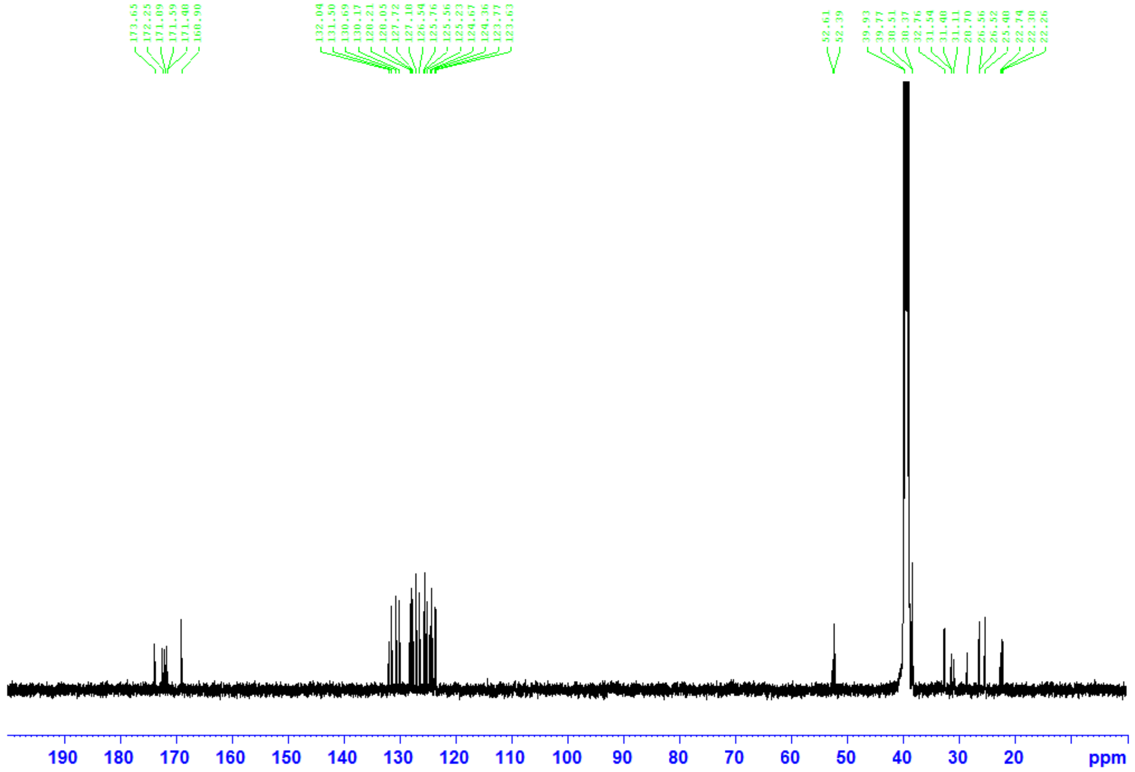

**MALDI-TOF MS spectra of AP 1:**

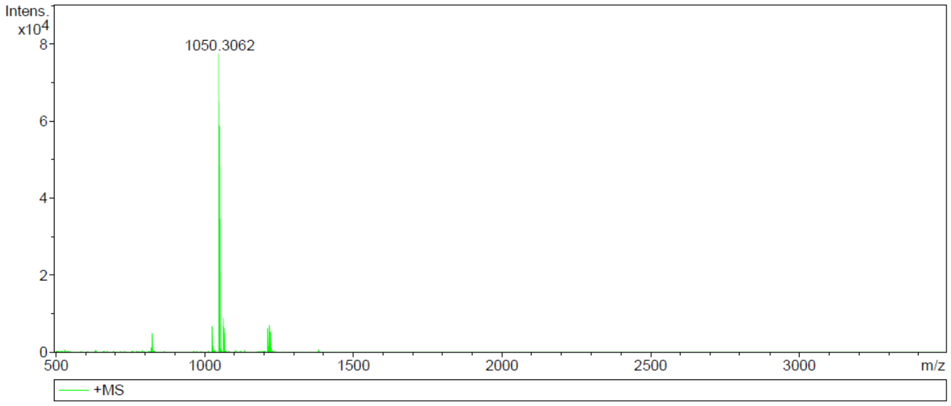


**Synthesis of CP:** Rink amide 4-methylbenzhydrylamine (MBHA) resin (0.6 mmol/g) was swollen in DCM and Fmoc-Lys(Boc)-OH was attached to the solid support under argon atmosphere with PyBOP as a coupling reagent in 5% DIPEA/DMF using three equivalents of each reactant. With the help of a single-mode microwave, the reaction mixture was irradiated for 20 min at 20 W and allowed to reach a maximum temperature of 60 °C. The coupling step was repeated to assure complete conversion of all accessible amino groups on the resin. After deprotection of the Fmoc group with 20% piperidine/DMF (1+5 min, 20 W, max. 60 °C), Fmoc-GABA-OH and pyrene carboxylic acid were coupled similarly using three equivalents of each reactant. Finally, the resin was thoroughly washed and dried and the receptor was cleaved from the solid support; the Boc-protected side chains were deprotected at the same time without microwave irradiation by utilizing a cleavage mixture composed of trifluoroacetic acid (TFA)/water/triisopropylsilane (TIS) (95:2.5:2.5). After purification by means of precipitation and reversed-phase medium-pressure liquid chromatography (MPLC) with 0.1% TFA in $H_2O$/MeOH and consequent anion exchange, probe CP was obtained as hydrochloride salt.

**Peptide CP:**

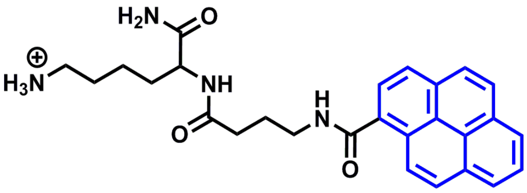

**Peptide CP** (26.4 mg, 57.6 μmol, Yield 48%, purity HPLC 99%). $^{1}$H NMR (500 MHz, DMSO-d$_6$): δ[PPM] 1.28-1.41 (m, 2H), 1.50-1.60 (m, 3H), 1.65-1.71 (m, 1H), 1.84-1.91 (m, 2H), 2.33 (t, *J* = 6.1 Hz, 2H), 2.73-2.77 (m, 2H), 3.37-3.47 (m, 2H), 4.22 (m, 1H), 7.01 (s, 1H), 7.42 (s, 1H), 7.90 (br, 3H), 8.04 (d, *J* = 6.8 Hz, 1H), 8.11-8.16 (m, 2H), 8.22-8.27 (m, 3H), 8.33-8.36 (m, 3H), 8.48 (d, *J* = 7.7 Hz, 1H), 8.75 (t, *J* = 4.6 Hz, 1H). $^{13}$C NMR (125.8 MHz, DMSO-d$_6$): δ[PPM] 22.44, 25.48, 26.62, 31.39, 32.81, 38.54, 52.16, 123.67, 123.80, 124.42, 124.72, 125.27, 125.60, 125.80, 126.60, 127.23, 127.74, 128.10, 128.25, 130.21, 130.74, 131.53, 132.13, 168.89, 171.90, 173.89. MALDI-TOF MS: cald 459.2391 for $C_{27}H_{31}N_4O_3^+$, found 459.2833.

**RP-HPLC chromatogram of Peptide CP:**

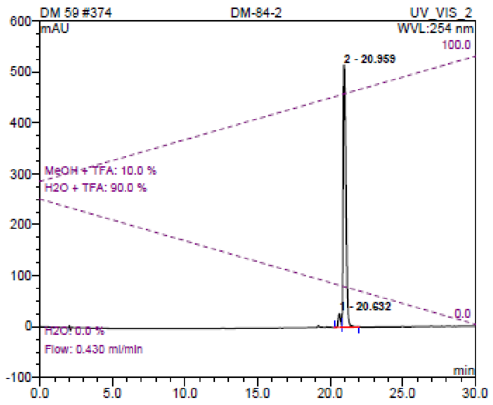


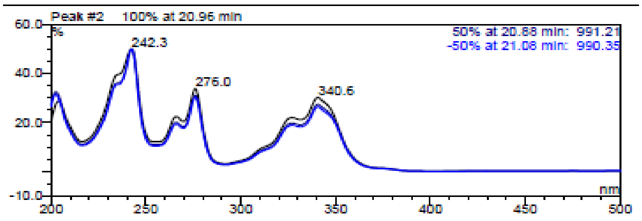

**500 MHz $^1$H NMR Bruker AVANCE 500 (DRX)**

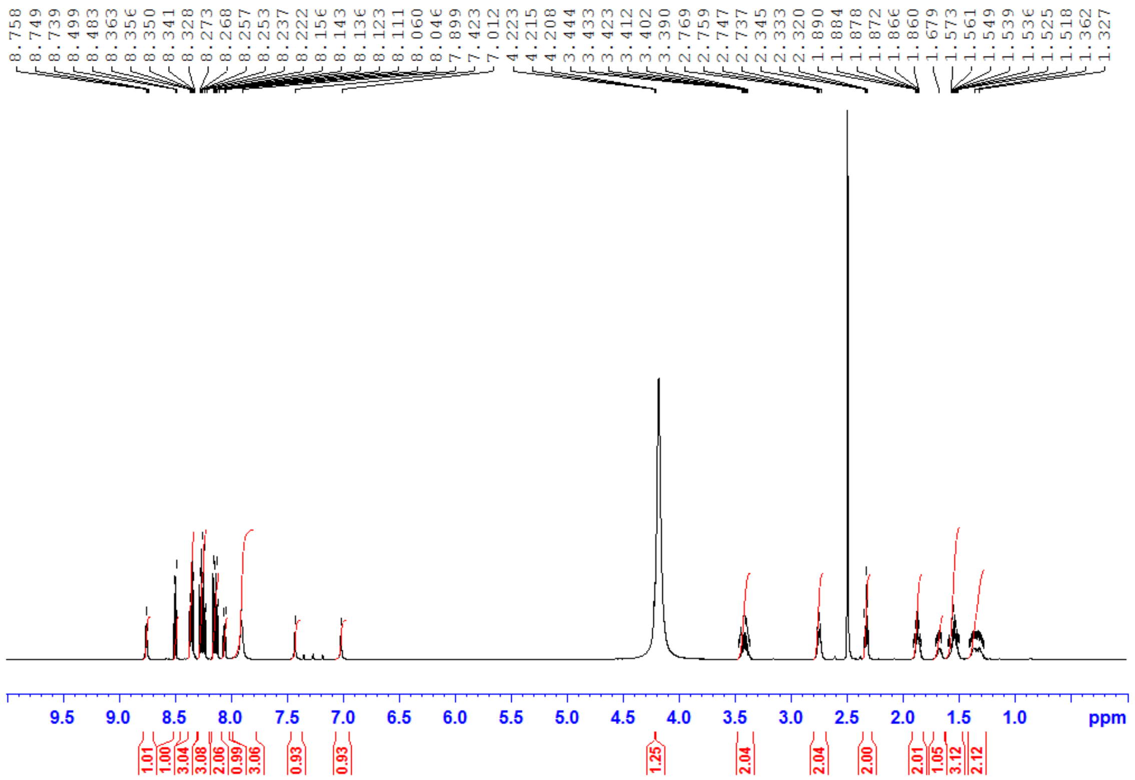

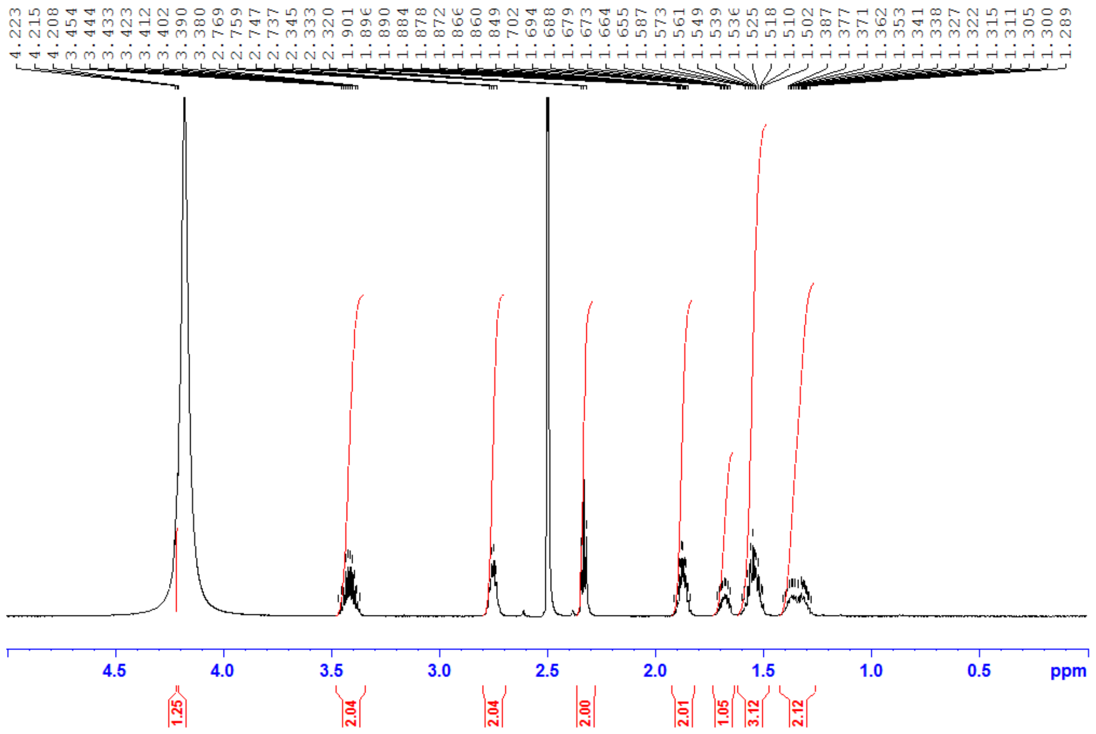

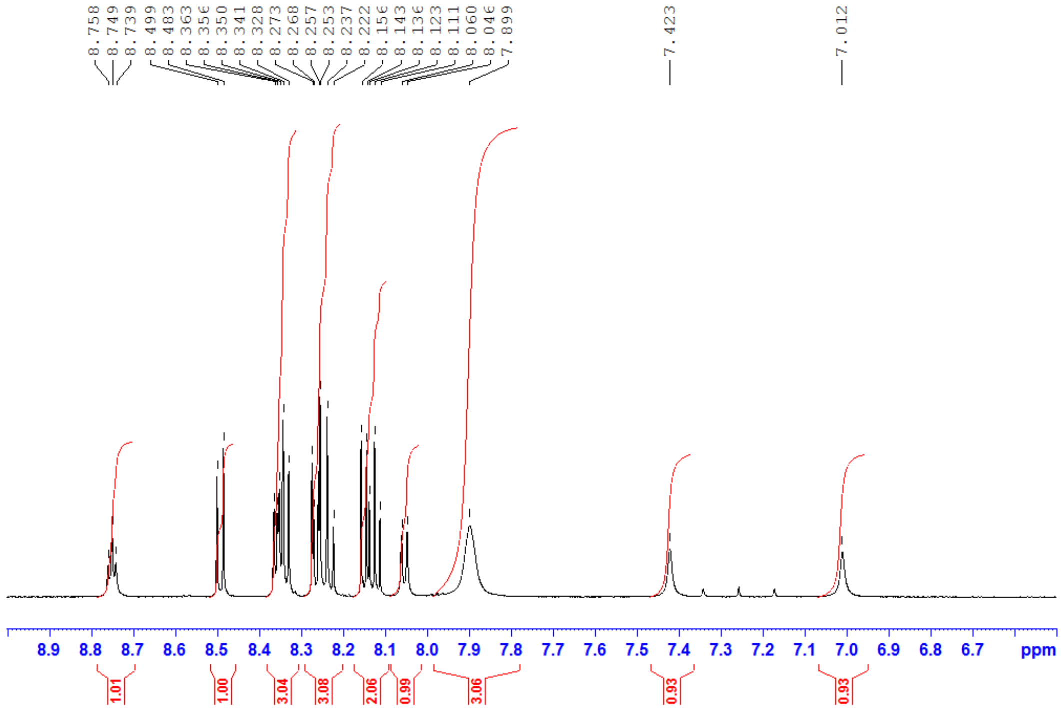

**125.8 MHz $^{13}$C NMR Bruker AVANCE 500 (DRX)**

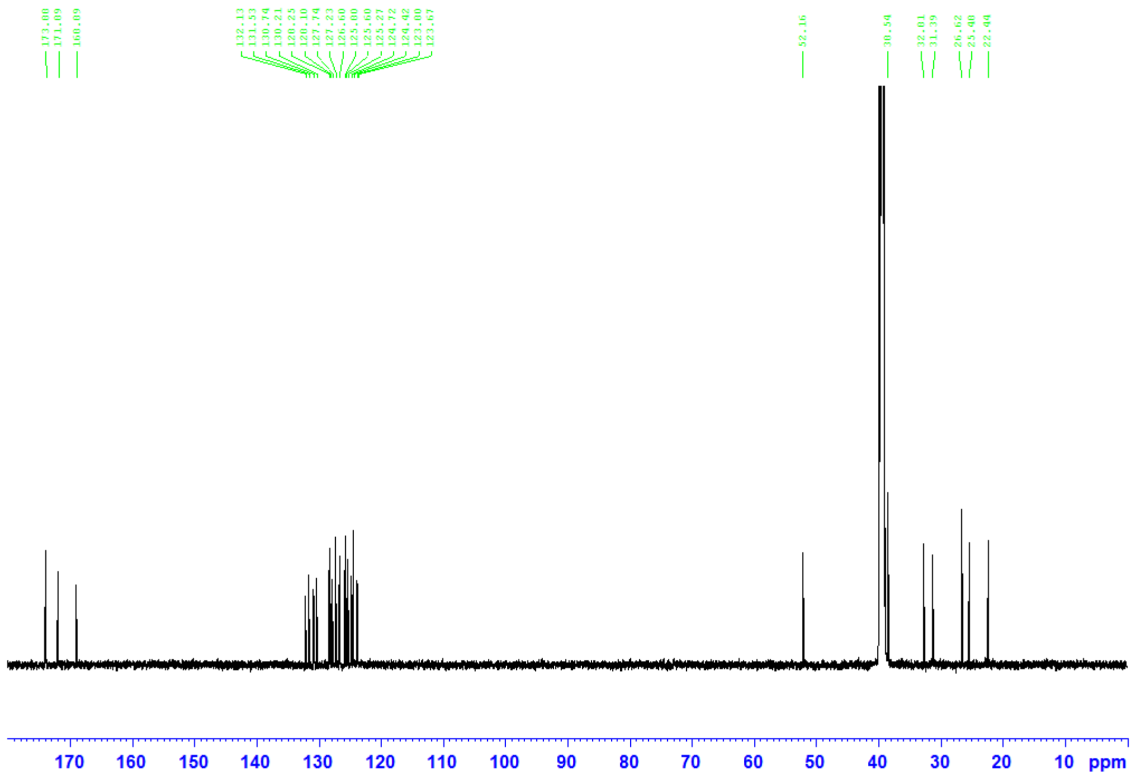

## MALDI-TOF MS spectra of CP:

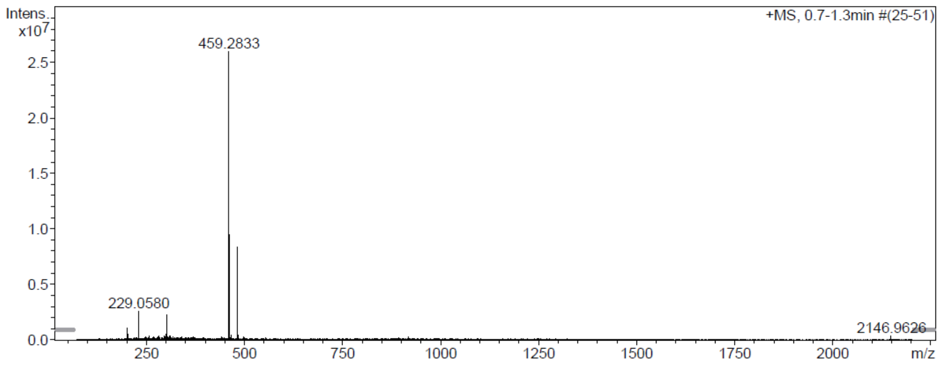


## 4. Additional experiments

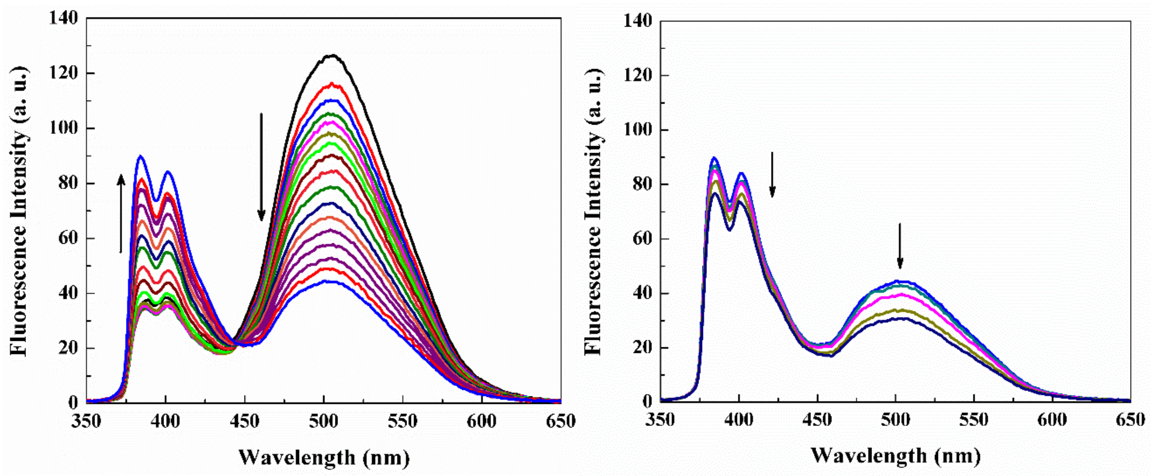


**Figure S1.** Fluorescence emission spectra of AP 1 (5 µM) upon addition of CB[8] (Left: 0 - 50 µM; right: 50-75 µM (excitation = 340 nm) in 10 mM HEPES buffer, pH = 7.4.

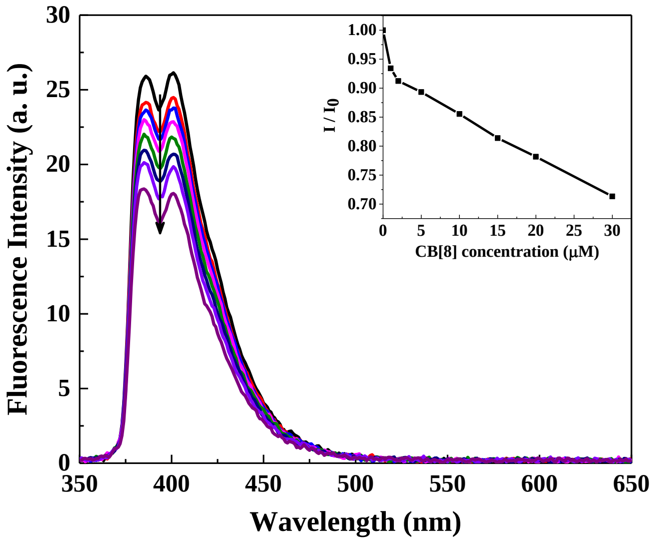


**Figure S2.** Fluorescence emission spectra of **CP** (5 μM) upon addition of CB[8] (0 - 30 μM) ($\lambda_{ex}$ = 340 nm) in 10 mM HEPES buffer, pH = 7.4. Inset: Relative fluorescence change during titration of **CP** by CB[8].

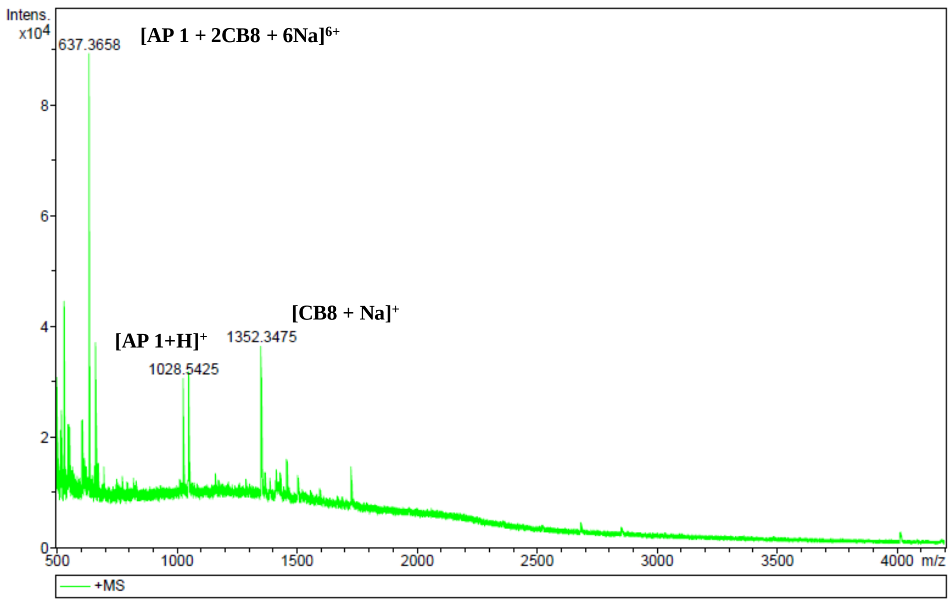


**Figure S3.** MALDI-MS spectrum of complex AP1•CB[8] in water.

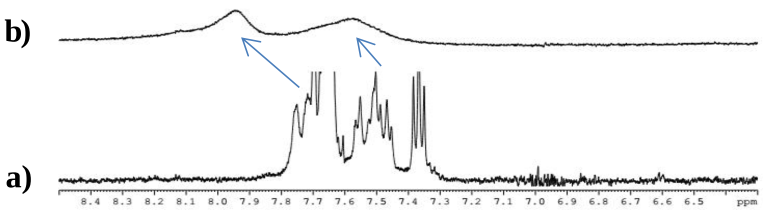


**Figure S4.** $^1$H NMR spectra in $D_2O$ at 25°C of a) peptide AP 1 (0.25 mM), b) AP 1 (0.25 mM) + CB[8] (0.5 mM).

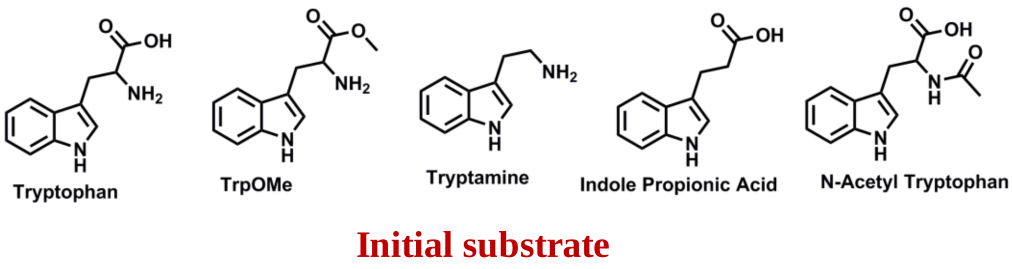


**Figure S5.** Structure of tryptophan derivatives as preliminary guests.

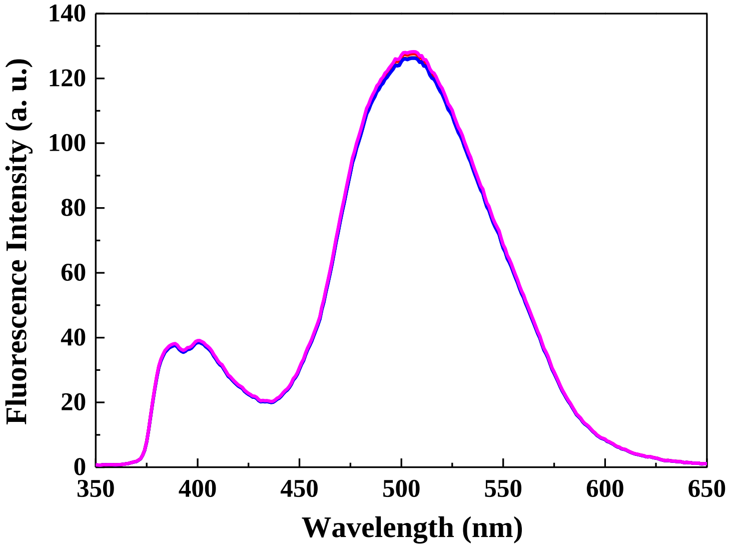


**Figure S6.** Fluorescence emission spectra of 5 µM **AP 1** upon addition of TrpOMe (0 - 50 µM) ($\lambda_{ex}$ = 340 nm) in absence of CB[8] in 10 mM HEPES buffer, pH = 7.4.

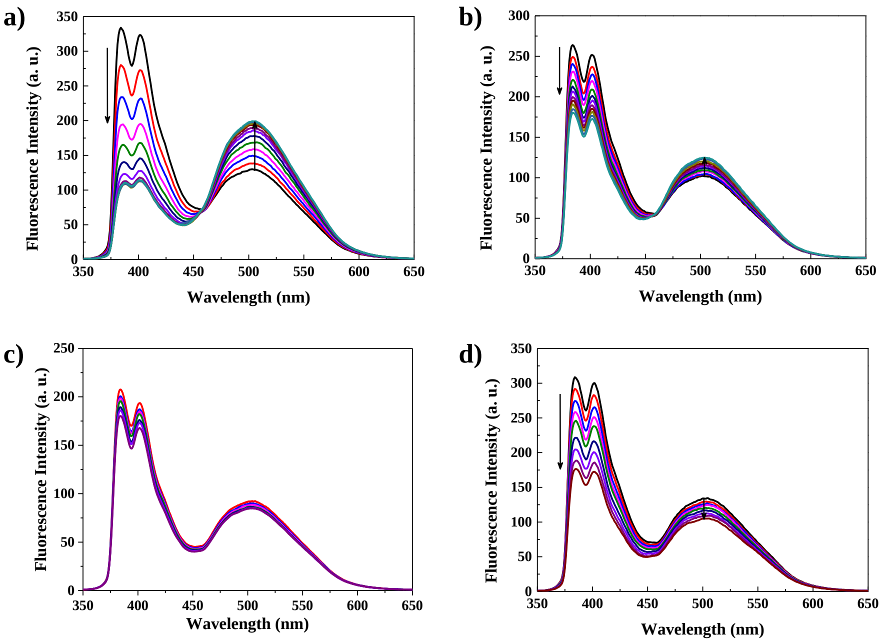


**Figure S7.** Fluorescence emission spectra of 5 µM **AP 1** and 50 µM CB[8] mixture upon addition of 0 - 55 µM of a) Trptamine, b) Indole priopionic acid, c) N-Acetyl tryptophan and d) Tryptophan respectively ($\lambda_{ex}$ = 340 nm) in 10 mM HEPES buffer, pH = 7.4.

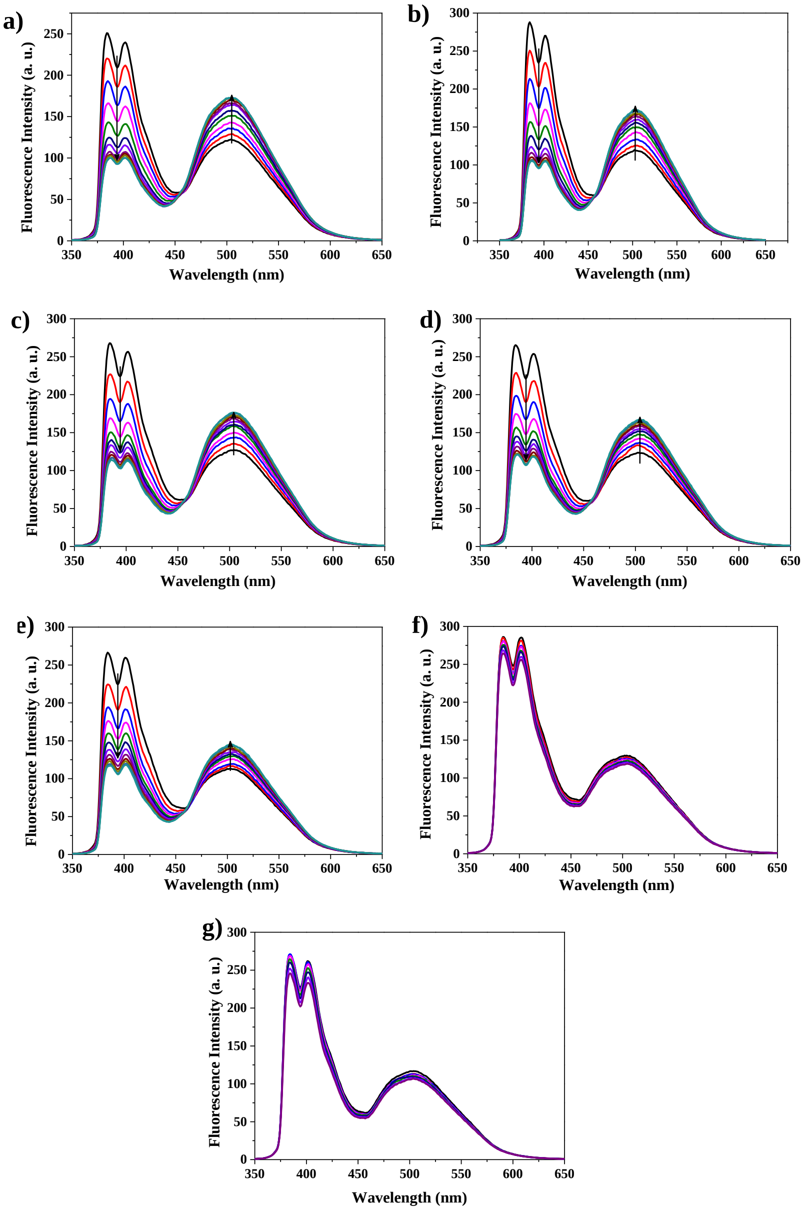


**Figure S8.** Fluorescence emission spectra of 5 µM **AP 1** and 50 µM CB[8] mixture upon addition of 0 - 55 µM of a) PhOMe•HCl, b) LeuOMe•HCl, c) LysOMe•HCl, d) ArgOMe•HCl, e) TyrOMe•HCl, f) AspOMe•HCl and g) GluOMe•HCl respectively ($\lambda_{ex}$ = 340 nm) in 10 mM HEPES buffer, pH = 7.4.

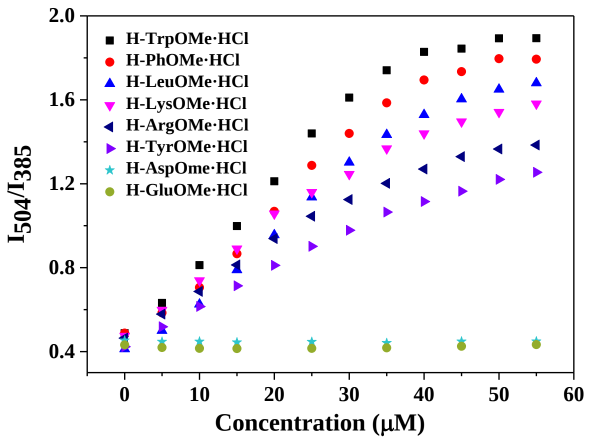


**Figure S9.** Ratiometric fluorescent responses of 5 μM **AP 1** and 50 μM CB[8] mixture upon addition of different amino acid methyl ester derivatives (0 - 55 μM) ($\lambda_{ex}$ = 340 nm) in 10 mM HEPES buffer, pH = 7.4.

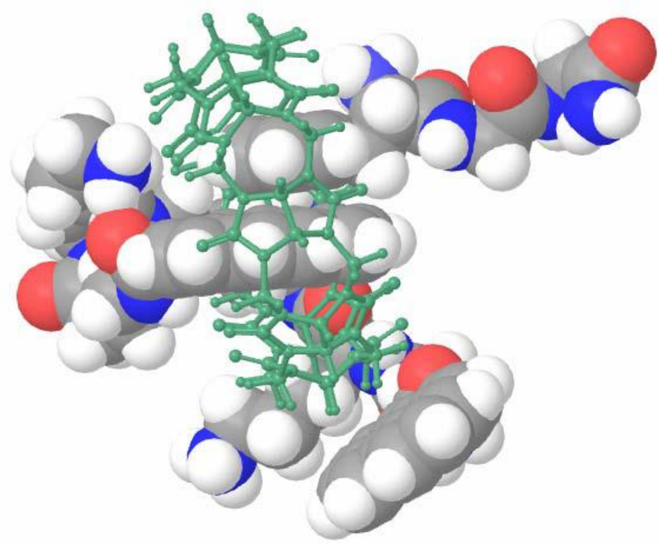

**Figure S10.** Molecular modeling showed that CB[8] cavity can accommodate one pyrene termini of AP 1 and N-terminal one phenylalanine residue of FGG together.

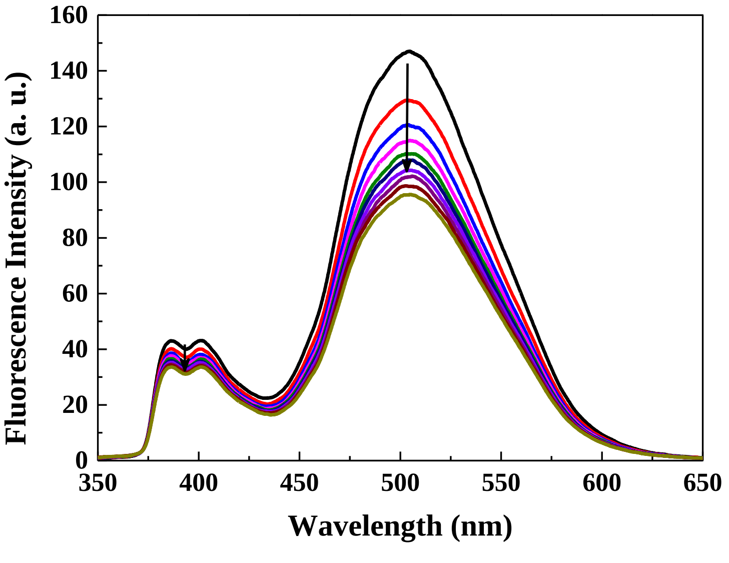


**Figure S11.** Fluorescence titration of 5 µM **AP 1** upon addition of FGG peptide (0 - 210 µM) in absence of CB[8] ($\lambda_{ex}$ = 340 nm) in 10 mM HEPES buffer, pH = 7.4.

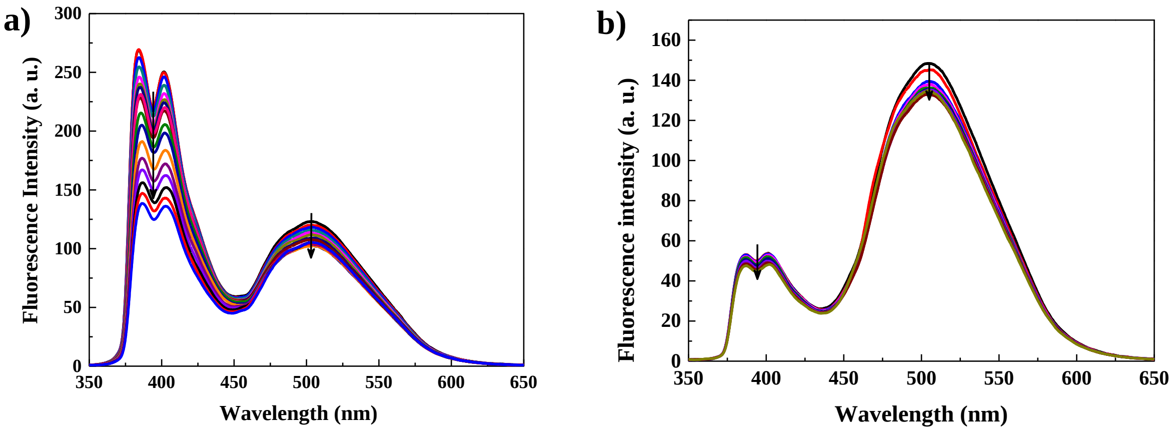


**Figure S12.** a) Full fluorescence titration of 5 µM **AP 1** and 50 µM CB[8] mixture upon addition of GFG peptide (0 - 210 µM) ($\lambda_{ex}$ = 340 nm) in 10 mM HEPES buffer, pH = 7.4; b) Fluorescence titration of 5 µM **AP 1** upon addition of GFG peptide (0 - 210 µM) in absence of CB[8] ($\lambda_{ex}$ = 340 nm) in 10 mM HEPES buffer, pH = 7.4.

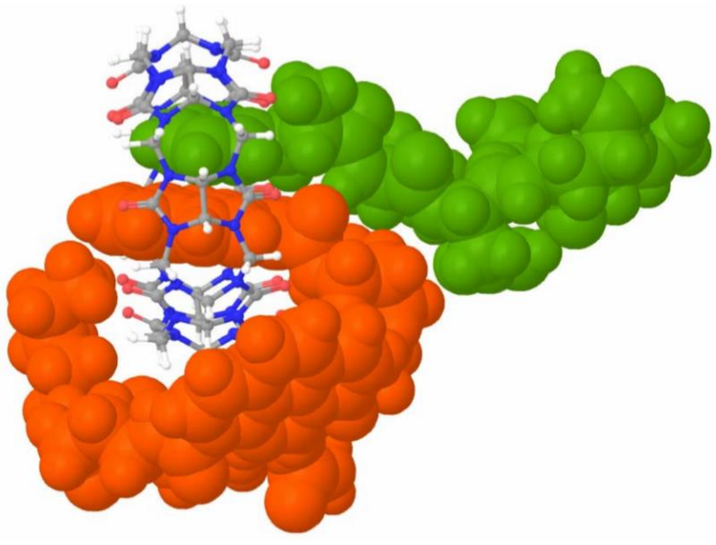

**Figure S13.** Molecular modeling showed that CB[8] cavity can accommodate one pyrene termini of AP 1 (orange) and N-terminal one phenylalanine residue (green) of insulin together.

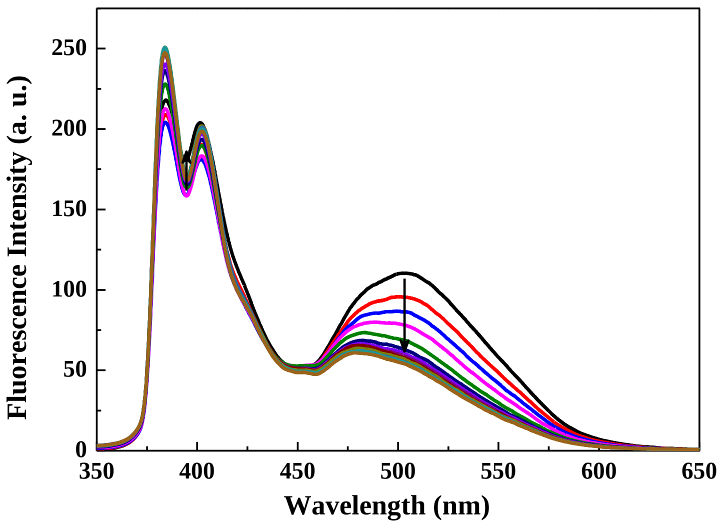


**Figure S14.** Fluorescence titration of 5 µM **AP 1** and 50 µM CB[8] mixture upon addition of BSA (0 - 20 µM) ($\lambda_{ex}$ = 340 nm) in 10 mM HEPES buffer, pH = 7.4.

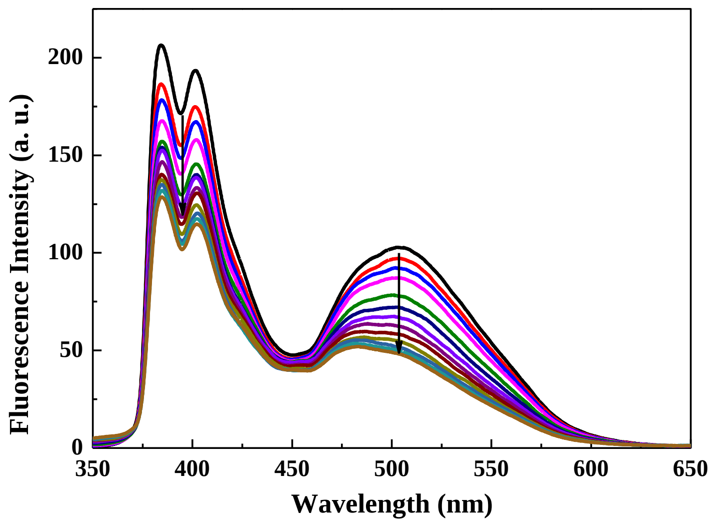


**Figure S15.** Fluorescence titration of 5 µM **AP 1** and 50 µM CB[8] mixture upon addition of BCA (0 - 19 µM) ($\lambda_{ex}$ = 340 nm) in 10 mM HEPES buffer, pH = 7.4.

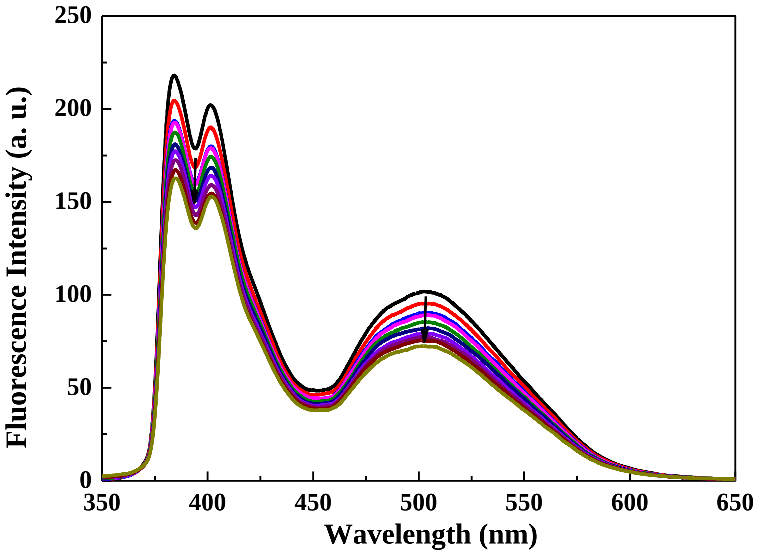


**Figure S16.** Fluorescence titration of 5 µM **AP 1** and 50 µM CB[8] mixture upon addition of IgG (0 - 13 µM) ($\lambda_{ex}$ = 340 nm) in 10 mM HEPES buffer, pH = 7.4.

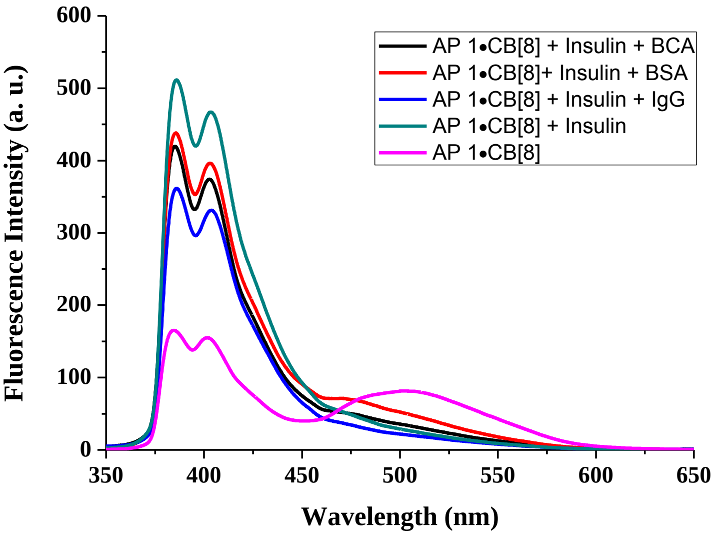


**Figure S17.** Competitive ratiometric fluorescence spectra for detection of insulin (20 µM) using 5 µM AP 1 and 50 µM CB[8] in presence of BSA, BCA, IgG respectively (20 µM) ($\lambda_{ex}$ = 340 nm) in 10 mM HEPES buffer, pH = 7.4.
